# Distribution of fitness effects of mutations obtained from a simple genetic regulatory network model

**DOI:** 10.1038/s41598-019-46401-7

**Published:** 2019-07-08

**Authors:** R. G. Brajesh, Dibyendu Dutta, Supreet Saini

**Affiliations:** Department of Chemical Engineering, Indian Institute of Technology Bombay Powai, Mumbai, 400 076 India

**Keywords:** Computational biology and bioinformatics, Evolutionary theory

## Abstract

Beneficial and deleterious mutations change an organism’s fitness but the distribution of these mutational effects on fitness are unknown. Several experimental, theoretical, and computational studies have explored this question but are limited because of experimental restrictions, or disconnect with physiology. Here we attempt to characterize the distribution of fitness effects (DFE) due to mutations in a cellular regulatory motif. We use a simple mathematical model to describe the dynamics of gene expression in the lactose utilization network, and use a cost-benefit framework to link the model output to fitness. We simulate mutations by changing model parameters and computing altered fitness to obtain the DFE. We find beneficial mutations distributed exponentially, but distribution of deleterious mutations seems far more complex. In addition, we find neither the starting fitness, nor the exact location on the fitness landscape, affecting these distributions qualitatively. Lastly, we quantify epistasis in our model and find that the distribution of epistatic effects remains qualitatively conserved across different locations on the fitness landscape. Overall, we present a first attempt at exploring the specific statistical features of the fitness landscape associated with a system, by using the specific mathematical model associated with it.

## Introduction

Mutations occur spontaneously during the course of reproduction of an organism. Mutations that impart a beneficial characteristic to the organism are selected and consequently, the frequency of the mutant allele increases in the population. Mutations can be single base changes called point mutations like substitutions, insertions, deletions, as well as gross changes like chromosome recombination, duplication, and translocation^1–5^. However, in a statistical sense, other than the point base substitutions, most other mutations are likely to cause drastic changes in fitness of the organism due to loss of function of affected genes, and hence are likely to be swiftly purified from the population.

In a framework where substitutions are the only possible mutations, the genotypic mutational space of the organism is fixed in size. This, in theory allows us to obtain the complete fitness landscape of the organism, by characterizing the fitness of every unique genotype, obtained by substituting all the possible combination of bases in the genome^6–8^. However, so vast is the scale of this complete venture, even for the tiniest of organisms like viruses or even one gene, that it becomes experimentally intractable. Hence, studies have limited to studying only small parts of the genome. For example, experiments have attempted to map the functional effect of mutations at important active site residues in proteins^9,10^, like Lunzer *et al*. engineered the IDMH enzyme to use NADP as cofactor instead of NAD, and obtain the fitness landscape in terms of the mutational steps^9^. Other experiments have attempted to ascertain how virulence is affected by mutations at certain important loci in viruses^11^. However, due to the scale of the genotypic mutational space, it has been extremely difficult to experimentally obtain fitness landscapes of larger multicomponent systems, and study the statistical properties of these landscapes like the Distribution of Fitness Effects (DFE). Attempts have also been made to back-calculate the underlying DFE by experimentally observing how frequently new beneficial mutations emerge and of what strength, but the final results were inconclusive^12^. As a result, how the beneficial, neutral, and deleterious mutations and their effects are distributed, when the organism genotype is at different locations on the fitness landscape, has remained largely intractable.

Since answering this question is experimentally intractable, it has been a focus of a number of theoretical investigations, which have explored the nature of fitness landscapes, and the corresponding DFEs. In 2003, Orr demonstrated the first approach to estimate DFE, using Gillespie’s mutational model, which uses Extreme Value Theory to estimate that, statistically, beneficial mutations should be exponentially distributed^13^. Satisfactory agreement with these predictions was found in a study where the authors study mutational effects in viruses^14^. Subsequent approaches use statistical analysis applied on drosophila population data and human amino acid mutations (or SNPs), to estimate DFE of deleterious mutations^15–17^. Approaches using population data assume that the present population is fitter with beneficial mutations enhanced in frequency, and thus, older variations must be lower in fitness. Minor variants that are declining in frequency are considered deleterious.

The above approaches, either assume an abstract mutational model, or use existing dynamic population level data to estimate fitness effect sizes of mutations (Amino Acid variants or SNPs) in the populations. These help provide a general picture, but cannot capture the specific dynamics of a real biological system. These limitations motivated us to ask if it would be possible to obtain a specific fitness landscape and DFE of a biological system, derived from the mathematical model defined to functionally characterize the system. Such mathematical models of biological systems when tuned with experimentally derived parameter sets, have been extremely successful in describing the behaviour and dynamical properties of a number of systems^18,19^. If these models truly capture the system’s mechanistic dynamics, it is expected that it should also be able to predict the change in the system dynamics upon change in the system parameters by way of mutations, and hence give us a handle to estimate the altered fitness of the organism.

**Figure 1:**
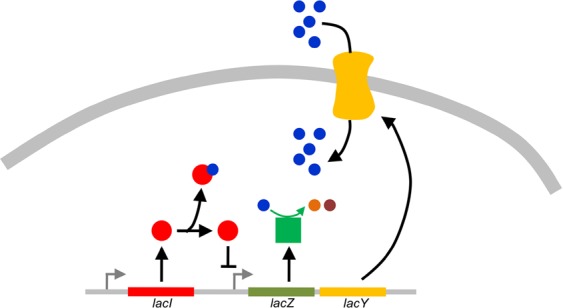
Schematic of *lac* operon regulation. In *E*. *coli*, the repressor protein LacI (red) negatively regulates transcription of the *lac* operon by binding to its operator site in absence of lactose. However, due to imperfect binding (leaky expression) small amounts of LacY protein (yellow) is produced, which imports lactose molecules (blue) into the cell, when present in the environment. LacI then preferably binds the lactose molecule and forms the LacI-lactose complex, which can no longer bind the operator site of the *lac* operon. This results in the transcription of the *lac* genes which produce the lac proteins LacZ and LacY (green and yellow, respectively). The latter further increases the import of lactose molecules, which is metabolized by LacZ (green) into glucose (orange), and galactose molecules (brown).

For this study, we chose the lactose utilization system in *E*. *coli*. The system is extremely well characterized^20–22^, and hence amenable to accurate mathematical modelling. Further, many models describing the system dynamics already exist^18,19,22^. Briefly, the *lac* operon system comprises of three genes, which are required for uptake and breakdown of lactose into simpler sugars (Fig. 1). These genes encode for a transporter LacY, a metabolic enzyme, LacZ, which metabolizes lactose into glucose and galactose, and a protein acetyltransferase LacA, which is believed to be involved in sugar metabolism via an unknown mechanism^23,24^. The expression of *lac* operon is regulated by a repressor protein, LacI^23–27^. In the absence of lactose, LacI binds the *lac* operon operator site and prevents transcription from the promoter. However, in presence of lactose, LacI preferentially binds a lactose molecule and thereafter is no longer able to bind the operator site of the *lac* operon, thus relieving the repression of the promoter. This makes transcription from the *lac* promoter conditional upon the presence of lactose in the environment^19,20,22,28–30^. In this study, we simulate the system in a defined environment with fixed lactose concentration. We compute fitness in terms of a simple cost-benefit framework using the steady state values of the system.

Using this framework, in addition to investigating the DFE associated with beneficial and deleterious mutations in the *lac* system, we seek to answer two specific questions. (1) How does the distribution of fitness effects change, with respect to the precise location on the fitness landscape? (2) How does this distribution vary between parameter sets associated with the network which correspond to the same fitness? To answer these questions, we choose parameter sets which correspond to low, medium and high fitness (with respect to global peak on the landscape) and introduce random mutations in the given parameter set, and note the fitness effect to build a frequency distribution associated with the parameter set. We also investigate the nature of epistatic effects between beneficial mutations using our model framework.

## Methods

### Model description

Lactose utilization in *E*. *coli* is enabled by the *lac* operon, which contains genes which encode for the sugar transporter LacY, the metabolic enzyme LacZ, and a protein LacA, which contributes towards lactose utilization via a yet to be characterized mechanism^31–33^. The expression from the *lac* promoter is controlled by the repressor protein LacI. In absence of lactose, LacI binds to the *lac* promoter and prevents transcription. However, when lactose is present, LacI preferentially binds the lactose molecule. The lactose-LacI complex can no longer bind the operator site of *lac* operon, thus relieving transcriptional repression, and resulting in transcriptional activation of the *lac* operon^20,22,28^. Mathematically, the dynamics of protein synthesis in the *lac* system Fig. 1 (parameters used to represent the system are listed in supplement Table 1) can be represented as following:

1 $$\frac{d[LacZ]}{dt}=Ba{s}_{1}+\frac{{K}^{y}}{{K}_{m}^{y}+[LacI]}-{k}_{d1}[LacZ]$$

2 $$\frac{d[LacY]}{dt}=(Ba{s}_{1}+\frac{{K}^{y}}{{K}_{m}^{y}+[LacI]})\times {K}_{t}-{k}_{d2}[LacY]$$

where $Ba{s}_{1}$ represents the basal activity from the *lac* promoter, resulting in expression of LacZ and LacY proteins, ${K}^{y}/{K}_{m}^{y}$ corresponds to the maximal promotor activity when LacI concentration is zero, ${K}_{m}^{y}$ is the half saturation rate constant associated with maximal promotor activity, and ${K}_{t}$ represents the translational capacity of LacY over LacZ^34^. The parameters ${k}_{d1}$ and ${k}_{d2}$ are degradation rate constants for LacY and LacZ proteins, respectively. In addition, dynamics of LacI concentration in the cell can be represented as follows:

3 $$\frac{d[Lac{I}_{tot}]}{dt}=Ba{s}_{2}-{k}_{d3}[Lac{I}_{tot}]$$

where $Ba{s}_{2}$ is the basal level expression from the *lacI* promotor, and ${k}_{d3}$ is the rate constant associated with LacI protein degradation.

At steady state $[Lac{I}_{tot}$] is evaluated as:

4 $$[Lac{I}_{tot}]=\frac{Ba{s}_{2}}{{k}_{d3}}$$

Free LacI is a decreasing function of intracellular lactose concentration $la{c}_{in}$, and can be evaluated as:

5 $$[lacI]=\frac{[lac{I}_{tot}]}{1+([la{c}_{in}]/K)}$$

where $[lac{I}_{tot}]$ is the total LacI concentration, and *K* is the half saturation constant for lactose-$la{c}_{in}$ binding.

The above equations can be used to approximate the dynamics of intracellular lactose inside the cell as follows:

6 $$\frac{d[la{c}_{in}]}{dt}=\frac{{k}_{1}[LacY][la{c}_{out}]}{{k}_{2}+[la{c}_{out}]}-\frac{{k}_{3}[LacZ][la{c}_{in}]}{{k}_{4}+[la{c}_{in}]}$$

where k_1_ and k_2_ are rate of influx of lactose and half saturation rate constant associated with lactose influx respectively; k_3_ and k_4_ are rate of efflux of lactose and half saturation rate constant associated with lactose efflux; and, k_5_ and k_6_ define the rate of metabolism of lactose and the half-saturation rate constant associated with lactose metabolism, respectively. The quantity $la{c}_{out}$ refers to the lactose concentration available in the surrounding media, and is equal to the lactose available to the cell for utilization. We treat the environment to be invariant, and hence the extracellular lactose concentrations is a constant.

### Cost-benefit framework

We use the above mathematical descriptions to define the fitness of the system in terms of a cost-benefit framework. The benefit conferred to the individual can be approximated as the amount of lactose metabolized per unit time at steady state and is quantified as:

7 $$Benefit=\frac{{k}_{3}[LacZ][la{c}_{in}]}{{k}_{4}+[la{c}_{in}]}$$

where $la{c}_{in}$ is the intracellular lactose.

The cost of the system can be approximated as proportional to the number of protein molecules that need to be produced per unit time to maintain the associated benefit^35^. Mathematically this can be represented as:

8 $$Cost=\alpha \times ({\alpha }_{z}\ast LacZ+{\alpha }_{y}\ast LacY+{\alpha }_{I}\ast LacI)$$

where, $\alpha$ is the cost per protein molecule; and ${\alpha }_{Z}$, ${\alpha }_{y}$, and ${\alpha }_{I}$ are the factors which estimate the cost (cellular resource expenditure) of producing LacZ, LacY, and LacI molecules needed to maintain their steady state levels. The three parameters are approximated as a product of their relative lengths (measured in number of amino acids) and the degradation constants associated with the three proteins^36^.

Eqs 1, 2 and 6 define a set of coupled Ordinary Differential Equations (ODE), which we solve numerically and compute the steady state values of each of the species. These steady-state values are then used to calculate benefit and cost. We define fitness of the system as the difference between the benefit and the cost.

### Selection of parameters and Mutational framework

This system, as defined above, will evolve towards higher fitness via one of the following mutations: (a) evolution of the protein to enhance performance of the enzymatic protein or the transporter or (b) optimizing the parameters that regulate the transcription of LacZ and LacY to best suit the environment of interest. Our simulations assume a short timescale of evolution where the likely relevant mutations are of type (b) and not type (a). In this study, we focus on mutations which control the regulation of the lactose system, and therefore, our mutational framework selects five specific parameters ($Ba{s}_{1},\,Ba{s}_{2},\,{K}^{y},\,{K}_{m}^{y},\,{K}_{t}$) that describe the transcriptional regulation in the cell. We keep all other biochemical parameters associated with the protein function constant.

Each parameter was allowed to take on values from a predefined range (Table S1). Thereafter, a mutation is introduced in one of the parameters of the system. The parameter that mutates was chosen randomly (with equal probability) from the set of five parameters, and a new value to the mutated parameter allotted. The new value was chosen randomly from a normal distribution centred on the original value of the parameter (we also performed simulations sampling from uniform distributions, and obtained qualitatively similar results). Thereafter, we simulate the network with the updated parameter set and calculate the associated fitness. We repeat the simulation process for the original parameter set for approximately 10,000 times to obtain a distribution of fitness effects due to mutations. These distributions of beneficial and deleterious mutations are then fitted to different types of theoretical distributions, and is finally represented by the distribution that best describes them (minimizes the Euclidean distance between the distribution and the simulation data).

We consider three distinct level of fitness as starting points for simulation of our network to obtain the nature of DFE: a low fitness (0.001 times maximum fitness possible in our model, f*max*), medium fitness (0.1 times f*max*), and high fitness (0.5 times f*max*). Also, since it is known that multiple parameter sets can correspond to the same fitness^35^, we identify multiple parameter sets, which correspond to fitness equal to the three starting levels (0.001f*max*, 0.1f*max*, and 0.5f*max*), to test how variable is the distribution of beneficial and deleterious mutations across multiple parameter sets.

### Studying epistasis

When the introduction of a mutation in two different backgrounds leads to two different fitness effect sizes, it is termed as epistasis. To observe the occurrence of epistasis in our model, we start with a parameter set **P0** (with parameter values {p1, p2, p3, p4, p5}, representing the 5 parameters we have considered free to mutate in our model framework) (Fig. S1), which corresponds to a fitness of 0.001*fmax* (*f0*). Next, we introduce a beneficial mutation *μ* (which changes the value of one of the parameters, say, p1 to p1*) to the set **P0**. The new set P0* = {p1*, p2, p3, p4, p5} corresponds to a fitness f0*. The net effect of this beneficial mutation on the fitness, is given by (f0* − *f0*), and is represented as Δ*f*. Next, we introduce a beneficial mutation (to set **P0**) in one of the parameters other than p1, say p2. The new value of p2 is p2_M_. The parameter set **P**_**M**_ = {p1, p2_M_, p3, p4, p5} corresponds to a fitness *f*_*M*_. Now, when the previous beneficial mutation *μ* in parameter p1 is again introduced into the set P_M_, it gives us **P**_M_* = {p1*, p2_M_, p3, p4, p5}. The corresponding fitness of this set is represented by *f*_M_*, and the benefit conferred by the mutation *μ* is represented by Δf* and is equal to (*f*_M_* − *f*_M_).

To quantify the impact of the genetic background on the effect of beneficial mutation *μ* (p1 → p1*) due to epistasis, this process was roughly repeated for four thousand distinct beneficial mutations spread over the rest of the parameters - p2, p3, p4 and p5. Similar analysis for parameters p2, p3, p4, p5 represents the same analysis done for the remaining parameters. The Supplementary Fig. S2 represents the entire analysis repeated four more times, by using different starting parameter sets with the same fitness 0.001*fmax*.

## Results

### Beneficial and deleterious mutations can be represented by exponential distributions

**Figure 2:**
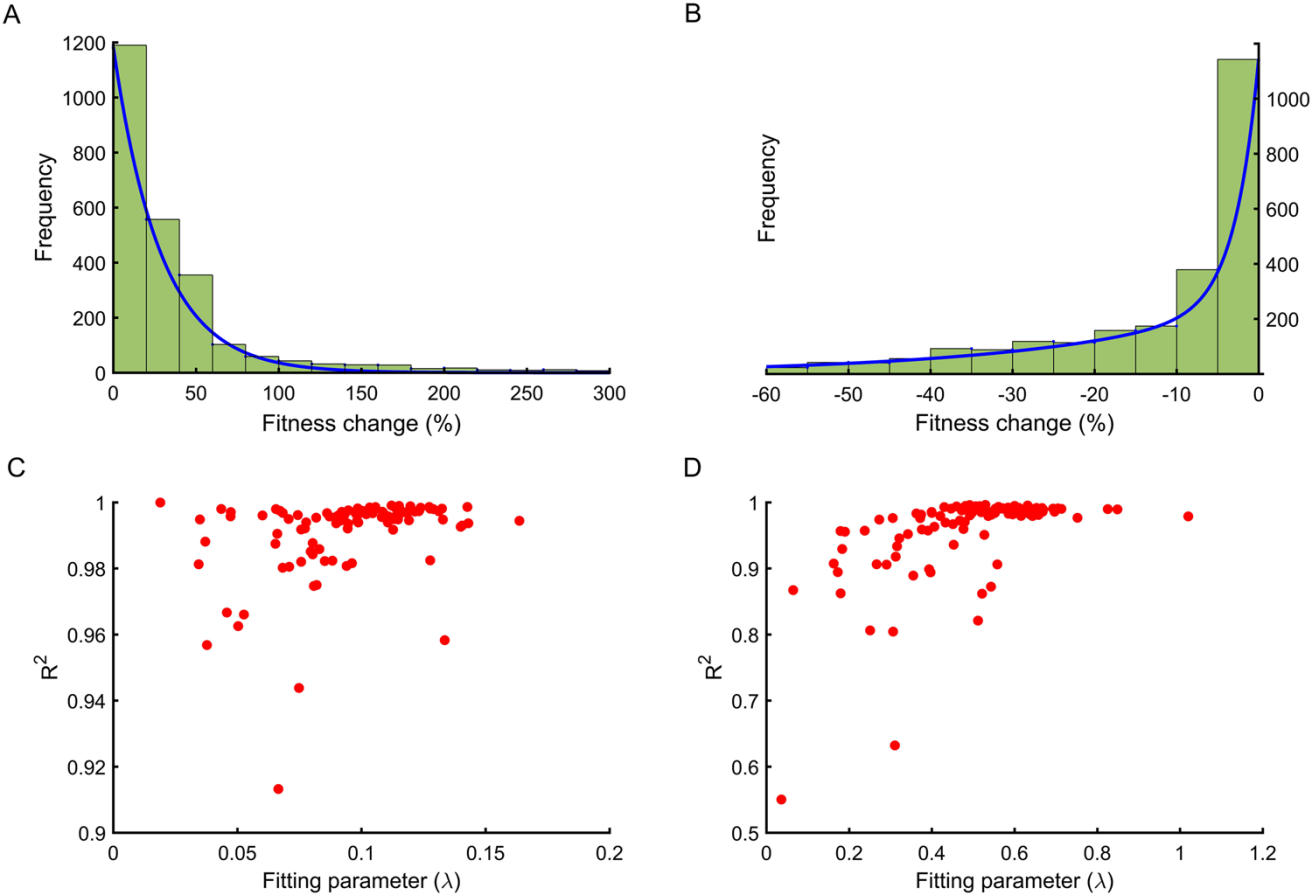
Beneficial and deleterious mutations can be represented as an exponential distribution at low system fitness. **(A)** Distribution of fitness effects of beneficial mutations when system fitness is 0.001f*max*. The distribution is fit well by exponential distribution with an R^2^ value of 0.9805. **(B)** Distribution of fitness effects of deleterious mutations when system fitness is 0.001f*max*. The distribution can be presented by exponential distribution with an R^2^ value of 0.9861. **(C)** 100 different parameter sets, corresponding to same 0.001*fmax* are simulated and DFE of beneficial mutations obtained as given in (**A**), the fitting parameter λ and goodness of fit R^2^ value is plotted. We note that beneficial mutations are fit well by exponential distribution irrespective of the precise location in the fitness landscape. **(D)** The DFE of deleterious mutations obtained from simulation of the 100 different parameter sets all with fitness 0.001*fmax*. The overall goodness of fit for deleterious mutations is significantly poorer compared to the beneficial mutations.

It has previously been shown that there is redundancy in the precise structure of genetic networks^35^, and that parameter spaces comprise a highly rugged landscape. The distribution of fitness effect of mutations likely depends on the precise location of the system on the fitness landscape. Hence, we identified the parameter sets that correspond to the same fitness value but have different parameter values. Thereafter, for each parameter set, a mutation was introduced and its effect on the fitness quantified. This process was repeated 10,000 times for each parameter set and the effects of these mutations used to obtain the distribution of fitness effects (DFE) for that particular parameter set. We plot the distribution of the beneficial and deleterious mutations separately. Figure 2A,B depict the distribution of beneficial and deleterious mutations respectively, for one such parameter set with a starting fitness value of 0.001*fmax*, when simulated for 10,000 mutations. We find that the distribution of the beneficial mutations was found to be best represented as an exponential distribution. The spread of deleterious mutations was also best represented using an exponential distribution in this case. From our simulations, we note that, for the starting fitness of 0.001*fmax*, 37.9% of mutations were beneficial and the remaining 62.1% of mutations were deleterious. While previous studies have explored and predicted distribution frequencies for mutations^11–13,37^, our framework gives us a handle to quantify and analyse these distributions in context of the physiological changes (represented by changes in particular parameter values) in the system.

Next, to explore the differences in the distributions of beneficial and deleterious mutations for parameter sets which are distinct and yet correspond to the same fitness, we repeated the above exercise for 100 distinct parameter sets (each corresponding to the same fitness), and compared it with the distribution we obtained in Fig. 2A. (The details of the parameter values used in the 100 sets are given in Table S2 in the Supplement). We found that while for all the hundred parameter sets, the distribution of beneficial and deleterious mutations can be represented as exponential distributions, significant differences in the quantitative nature of the distributions exist. To demonstrate the same, we represent the parameter characterizing the exponential distribution and the associated R^2^-value obtained from the goodness of fit for these hundred parameter sets. Our results show that while the statistical fit (${R}^{2}$) associated with the representation of these distributions as an exponential distribution is very good, the precise parameter value ($\lambda$) of the exponential, which describes a particular parameter set, is variable in nature (Fig. 2C,D). These results show that while the local structure of the fitness landscape perhaps does not influence the frequency distribution of mutations qualitatively, the precise quantitative nature of mutations is significantly influenced by the local environment on the fitness landscape. Additionally, comparing Fig. 2C,D, we can see that while the distribution of deleterious mutations (for a starting fitness of 0.001*fmax*) can be represented using an exponential distribution, but the quality of fit for this distribution is not as good as that for beneficial mutations.

### Effect of the starting system fitness on the frequency distribution of beneficial and deleterious mutations

We already demonstrated that DFEs can be qualitatively same but quantitatively different based on the specific parameter set even though all of them exhibit the same fitness. Next, we test the impact on the nature of DFE based on starting parameter sets of different fitness. Our naïve expectation regarding this exercise is that as we move towards a local or global peak on a fitness landscape, two features would be observed. (1) The percent of beneficial mutations available to a network should decrease, and this trend should be observable through the framework we use in this study. (2) The qualitative nature of distributions of fitness effects of mutations should be independent of the fitness corresponding to the parameter set we start with. To test these predictions, we perform simulations in the same way as performed in the previous section. In these cases, the chosen starting parameter set corresponded to (a) 0.001f*max*, (b) 0.1f*max*, and (c) 0.5f*max*. Again, just as in the previous section, we choose a set of hundred parameters corresponding to fitness levels 0.01 or 0.1 times f*max*. For the fitness level 0.5f*max*, we could identify only 80 parameter sets (presumably because parameter sets corresponding to higher fitness values become increasingly sparse).

**Figure 3:**
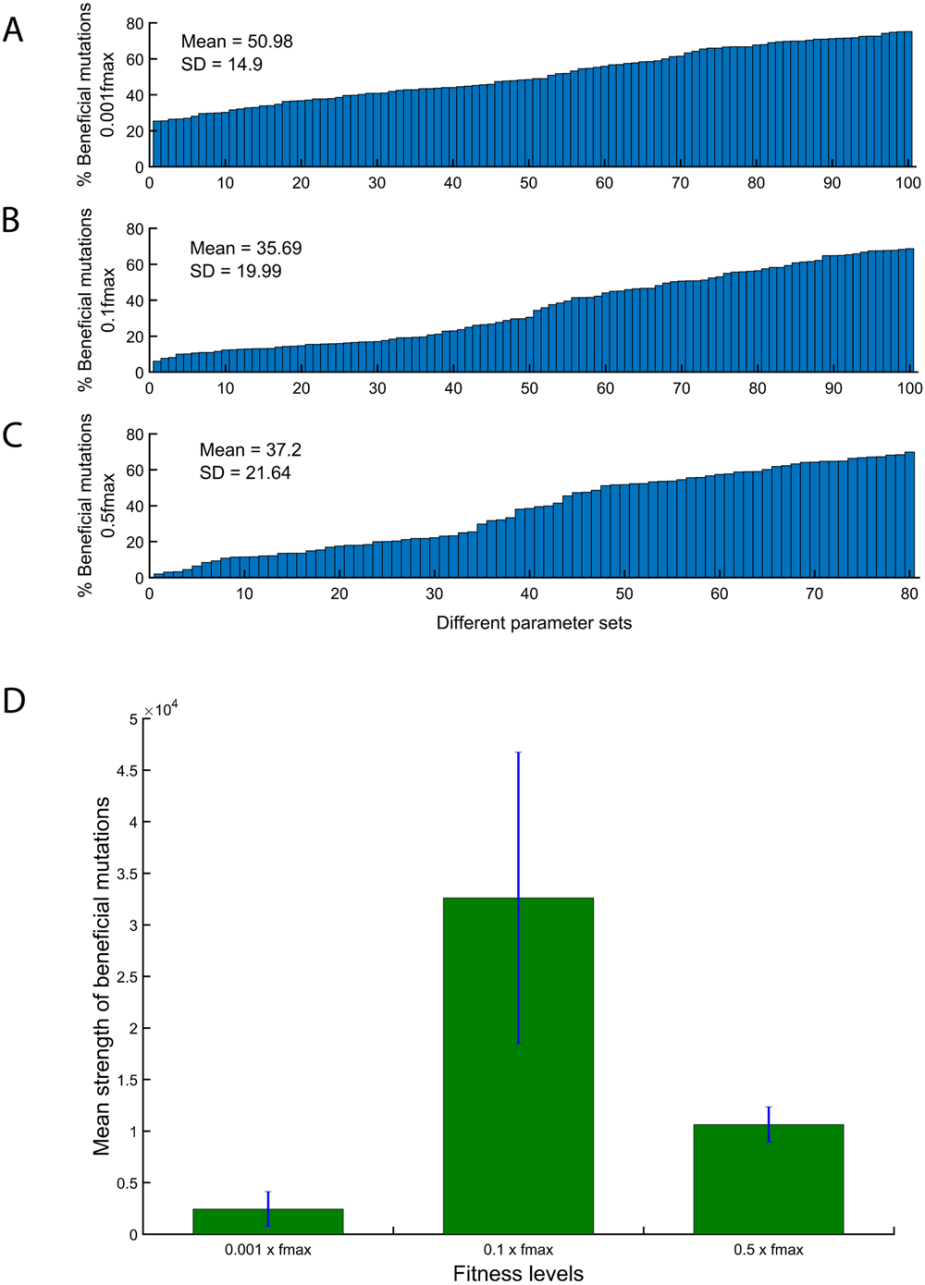
Changes in distribution of beneficial mutations with changes in the fitness. The percent of beneficial mutations (of all mutations introduced) decreases as the fitness corresponding to the initial parameter set increases: **(A)** 100 parameter sets corresponding to fitness 0.001*fmax*, **(B)** 100 parameter sets corresponding to fitness 0.1*fmax*, and **(C)** 80 parameter sets corresponding to fitness 0.5*fmax*. The variation in percent beneficial mutations between different sets increases significantly as the initial fitness increases. **(D)** The mean fitness effect of the beneficial mutations peak at intermediate values of initial fitness.

Consistent with our hypothesis, we note that as the fitness of the system as defined by the initial parameter set increases, the percent of beneficial mutations decreases **(**Fig. 3A–C). Further, two observations stand out. The rate of decrease in the percent beneficial mutations decreases as the fitness increases, and secondly, the variation in the percent of beneficial mutation corresponding to a particular parameter set increases. Given our intuitive understanding of fitness landscapes, perhaps these results are not surprising. As we move towards higher fitness on a landscape, the fractions of mutations which are beneficial decrease.

In addition, the mean effect of a beneficial mutations is small in parameter sets corresponding to low fitness; is maximum for parameter sets corresponding to intermediate fitness; and is low again for parameters corresponding to high fitness (Fig. 3D). Our model does not explicitly explain this non-monotonicity in fitness effect with respect to the fitness of the background. A plausible explanation could be that parameter sets corresponding to low fitness are likely in the ‘valley’ of the multidimensional fitness landscape. The parameter sets corresponding to intermediate fitness are along the slope of the fitness peak; and those corresponding to high fitness have limited availability of beneficial mutations to reach the peak. We do not, however, discount that this result could be an artefact of modelling and/or due to the numerical values used to describe fitness. These two effects, either in isolation or combined, consequently explain the results and trends presented in Fig. 3D.

**Figure 4:**
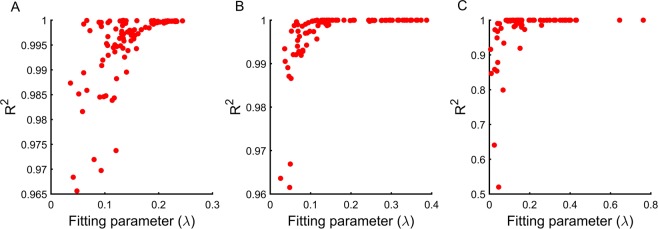
DFE of beneficial mutations can be represented by exponential distribution for all three initial fitness. System is simulated for (**A**) 0.01*fmax*, (**B**) 0.1*fmax*, and (**C**) 0.5*fmax* with 100 different parameter sets (80 parameter sets for 0.5*fmax*). Frequency distribution of beneficial mutations associated with all three fitness levels can be represented by exponential distribution. The fitting parameter (λ) and R-square associated with each parameter sets are plotted. We see that, when system is at low fitness level (0.01*fmax* and 0.1*fmax*) the exponential distribution can fit the data accurately (high R-square), in case of high fitness (0.5*fmax*) however, exponential distributions do not fit as accurately (low R-square).

**Figure 5:**
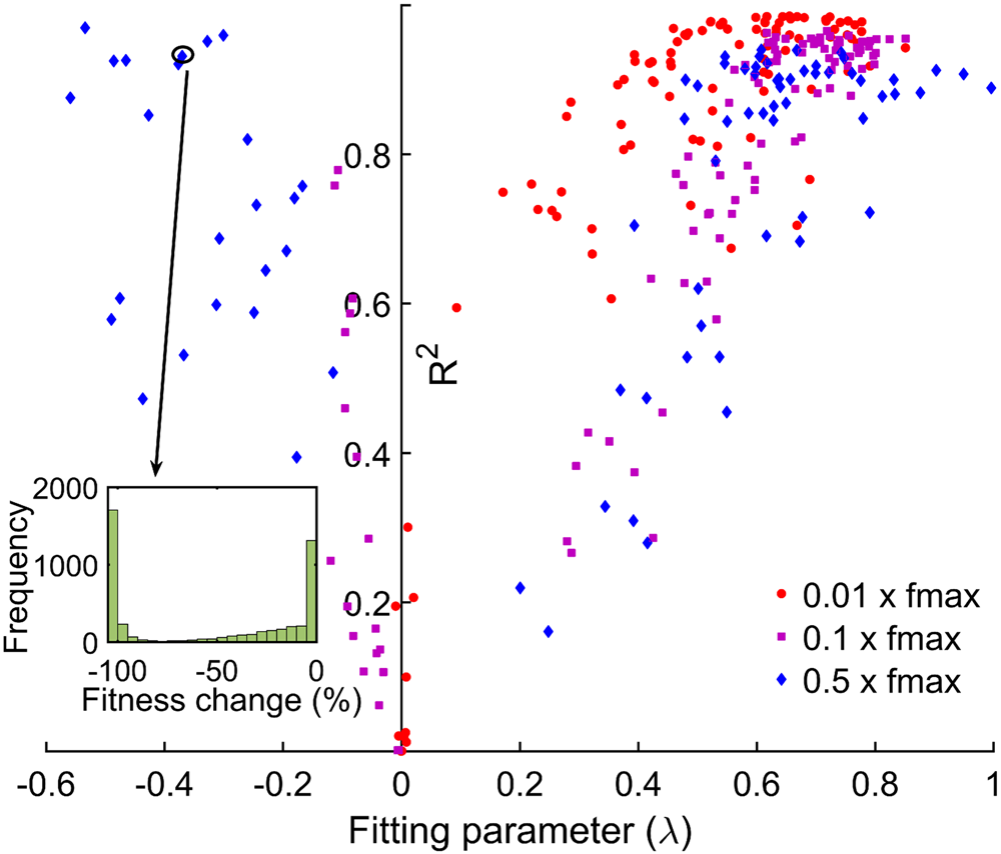
DFE of deleterious mutations cannot be represented by any standard distribution for all three initial fitness. System is simulated for 0.01fmax, 0.1fmax and 0.5fmax with 100 different parameter sets (80 parameter sets for 0.5fmax). DFE of deleterious mutations associated with all three fitness levels cannot be represented any standard distribution. The fitting parameter λ and goodness of fit ${R}^{2}$ associated with each parameter sets are plotted. We see that the overall quality of fit to exponential distribution is poor for all three initial fitness. In addition, we also note that for many parameters deleterious frequency distribution have two-peak (inset).

When we analyse our results for DFE of beneficial mutations, we note that the spread of beneficial mutations can be represented by an exponential distribution for all three fitness levels (fitness equal to 0.01, 0.1 or 0.5 times *fmax*) (Fig. 4). Our results show that while these distributions are represented using exponential distributions with a high level of statistical accuracy for most parameter sets, at high starting fitness, the DFE of beneficial mutations in a few parameter sets are poorly approximated by exponential distributions. The same is true for deleterious mutations. However, while at lower fitness levels the distributions of deleterious mutations are aptly represented by exponential distributions, the same does not hold for many parameter sets corresponding to higher fitness (Fig. 5). The fraction of parameter sets that cannot be suitably represented by an exponential distribution increases with increasing starting fitness. These sets are best represented by a negative value of the fit parameter of the exponential distribution. To explore the reason for this distribution, we plot the raw frequency data for each of these distributions. For many of the parameter sets, the distributions of the deleterious mutations were two-peaked. The first peak of the distribution corresponds to the mutations which are weakly deleterious in nature. The second peak corresponds to large deleterious effects of mutations. These two-peaked DFE for deleterious mutations have been previously anticipated in literature^38^.

### Epistatic interactions between beneficial mutations

Our study also aims to study how the fitness effect of beneficial mutations change when they are acquired in different genetic backgrounds, i.e. study the effect and extent of epistasis between beneficial mutations, in the context of lactose utilization in *E*. *coli*. For this purpose, we compared the difference in fitness gained, when the same mutation (change in parameter value) is acquired by a parameter set alone (Δ*f*) or in conjunction with another beneficial mutation (Δf*) in another parameter. (See Methods for details)

**Figure 6:**
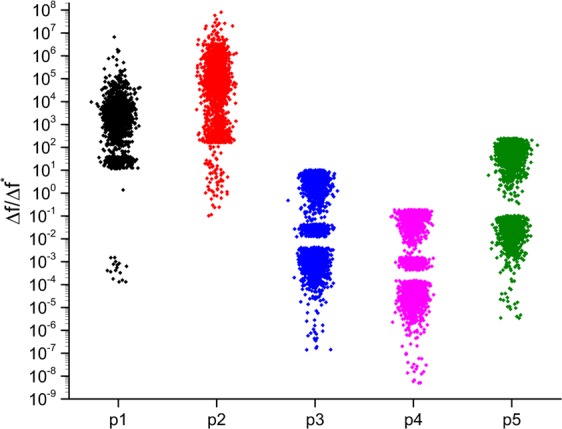
Epistatic interactions between beneficial mutations. Beneficial mutation in a specific parameter pi (x-axis) is introduced into two backgrounds: one where there is no other beneficial mutation and another where the parameter set is carrying another beneficial mutation in pj. The ratios of benefit conferred by pi in the two sets is plotted (∆f corresponds to benefit conferred by pi in the original set; and ∆f* refers to the benefit conferred in the set carrying the beneficial mutation pj). For each mutation pi, around 4000 sets carrying a distinct beneficial mutations (pj-s) are used. A ratio of more than one implies greater benefit being conferred by pi in the original set than in the presence of the beneficial mutation pj. For details on the clusters for each parameter represented on the X-axis, see Supplement Fig. 2.

In Fig. 6, we represent the distribution of the ratio Δ*f*/Δf* for about 4000 parameter sets, in the Y-axis and the X-axis represents the parameter, change in which represents the beneficial mutation, corresponding to which the ratio is being calculated. From the Figure, we can see that the resultant effect of a beneficial mutation can be quite drastically different in different parameter backgrounds. For example, for the mutation in parameter p1, the benefit of the mutation is 10 to 10^5^-fold lower in the sets **P1**-**P4000**, compared to the original set **P0**. Interestingly, p2 shows a qualitatively identical trend as well. For parameters p3 and p4, the trend is reversed (compared to **P0**, the newer sets exhibiting higher benefit conferred by the mutation). The beneficial mutation corresponding to parameter p5 is almost evenly spread where it confers a higher benefit to **P0** compared to around 2000 sets; and confers a higher benefit to the other 2000 sets (compared to **P0**). Interestingly, these results hold independent of the precise mutation in question. As shown in Supplement Fig. 2A–D, four distinct mutations in each of the parameters were introduced and the same analysis performed on each. The qualitative nature of the distribution of the ratio ∆f/∆f* does not change with the precise mutation introduced.

For each parameter, we see points distributed in distinct clusters in the Fig. 6. Further analysis revealed that they correspond to background mutations in the same parameter that is introduced in the set **P0** (to make it **P**_**M**_), which suggests an intrinsic relationship between parameters, when defining the fitness corresponding to a set^39^. This is illustrated in Supplement Fig. S3. The key idea being that there is a significant amount of variation in the effect of a mutation, and this is strongly dependent on not only the starting fitness corresponding to the parameter set but also the precise values of the associated mutated parameters.

**Figure 7:**
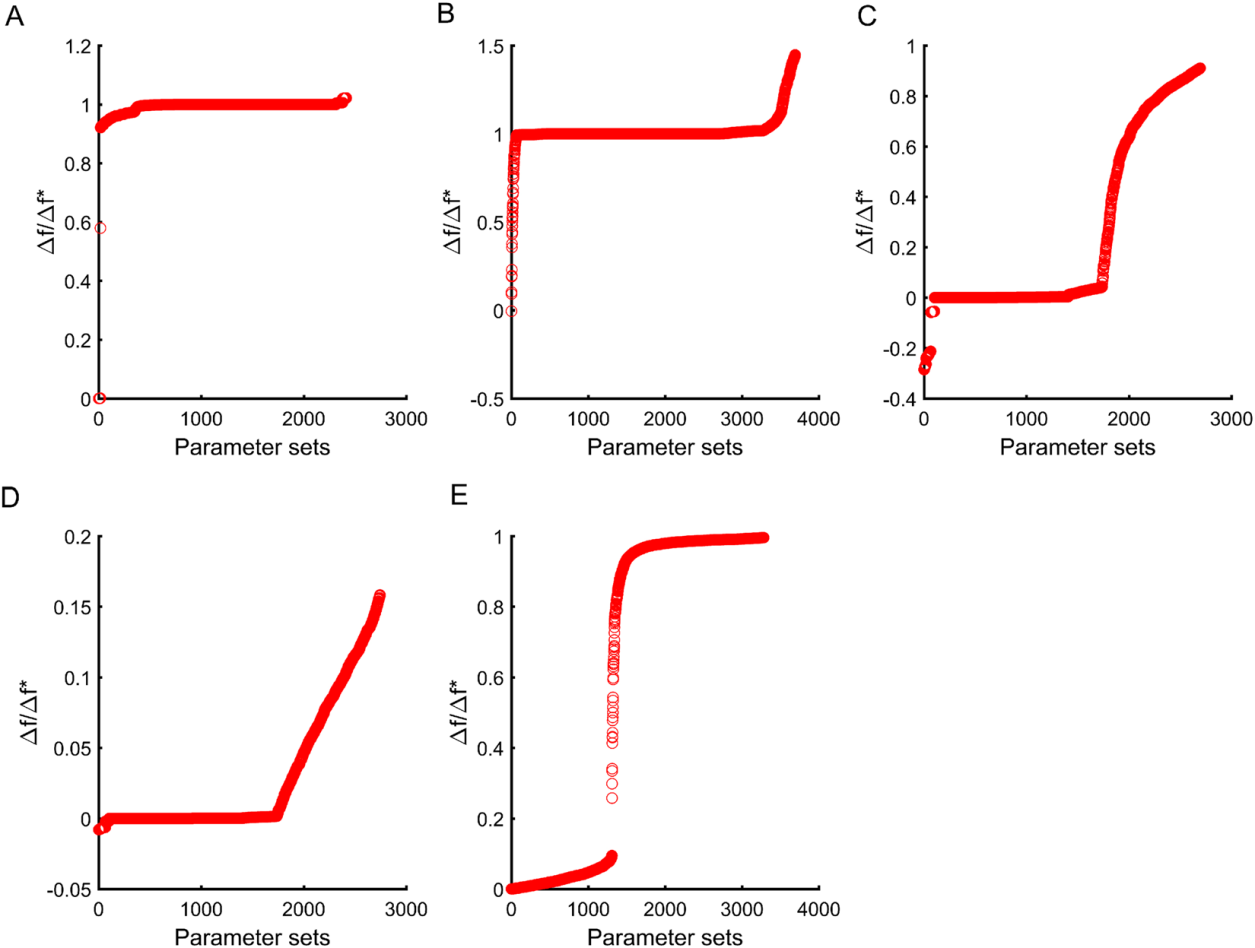
Magnitude of fitness effect of beneficial mutations decrease with increasing fitness. We plot the ratio between the differences in fitness, when the same mutation is acquired alone by one parameter set (Δ*f*) versus when in conjunction with another beneficial mutation in another parameter (Δf*) (Y-axis), across different parameter sets arranged in the increasing order of their fitness (X-axis). The parameter sets are generated the same way as discussed for Fig. 6. (**A**) refers to the sets when the beneficial mutation is introduced in parameter p1 and the sets on the X-axis are carrying a beneficial mutation in a parameter other than p1. **(B–E)** refer to sets when the beneficial mutation is introduced in parameters p2, p3, p4, and p5. Parameters p1, p2, p3, p4, and p5 are the same as described before.

To test another aspect of this epistasis between beneficial mutations, we arranged the parameter sets (all the **P**_**M**_ sets) in increasing order of their starting fitness. This is represented by the X-axis in Fig. 7, and on the Y-axis, we represent the ratio Δ*f*/Δf*. We observe that for a beneficial mutation corresponding to each of the five parameters, the resultant curve is a strictly increasing function. This shows that the benefit conferred by a beneficial mutation decreases with increasing fitness corresponding to the parameter set that it was introduced in. Again, the trends, as shown in Fig. 7, are not dependent on the precise mutation in question. We repeat the analysis for five different mutations (for each parameter), and observe qualitatively identical trends (Supplement Fig. S4). Interestingly, a small fraction of parameter sets exhibit sign-epistasis, where the beneficial mutation in the original set leads to reduction in fitness in the set **P**_**M**_. These sets exist when the mutation *μ* is introduced in p3 or p4. This is found to happen for a limited parameter sets when the context of the two beneficial mutations is different. More specifically, this sign-epistasis is observed when the benefit conferred by one mutation increases benefit (by increasing LacZ production) and the other benefical mutation reduces cost (for instance, by decreasing the production of LacY).

## Discussion

While knowledge of the fitness landscape can help us understand the evolutionary trajectory of an organism, determining the same remains experimentally intractable. Hence, we investigated the possibility of extracting statistical properties like DFEs of the fitness landscape for a particular biological system, using its associated mathematical model. In this work, we developed a quantitative framework to obtain these distributions in the context of a specific genetic network: the lactose utilization system in *E*. *coli*, as it is grown in a constant environment with high lactose concentration. In addition to, asking what the nature of the distributions of beneficial and deleterious mutations were, we wanted to understand if it was dependent on the specific location on the fitness landscape, or dependent on the initial fitness of the system or both. While the assumption of growth in a constant lactose environment is likely physiologically unrealistic, it represents growth of bacteria in a chemostat in laboratory environments. Moreover, our analysis can be extended to time varying concentrations of lactose, without introduction of any additional conceptual details.

Our results show that beneficial mutations are well-represented by exponential distributions. However, it is much more difficult to comment on the distribution of deleterious mutations. We also characterized these distributions as we moved towards higher fitness (climbed a fitness peak), or moved on the landscape to another location with the same fitness.

Fitness landscapes not only depend upon the genotype of the organism but the interaction between the phenotype and the environment. Thus, the fitness landscape can change completely in every unique environment. Here, we limited our analysis to the simple case of a constant high lactose containing environment where the lac system exhibits no multi-stability^18,22^ and hence allows for our modelling assumptions to be true. While constant high lactose concentrations are not ecologically relevant environmental niches, they do reflect the kind of constant nutrient-rich environments in laboratory setups, and are hence are relevant.

Our analytical approach can be extended to any system whose role in the physiology of the organism is clear. If one cannot precisely model the molecular mechanisms involved in the given system, and instead uses empirical fit parameters, our approach will cease to provide accurate readouts of fitness, when the parameters are altered too far from their experimentally calibrated values. Furthermore, the mathematical model should be developed to only contain parameters that are independent of each other, in terms of effect of mutations. Else, one will be unable to predict the corresponding change in the coupled parameters, due to mutation of one, and hence be unable to account the respective changes in the fitness. For example: in the lac system, a mutation in the lac operon may alter both the lacZ and lacY protein production together because they share a common promoter. Hence, in our model we choose to have the same production parameters for both ($Ba{s}_{1},\,{K}^{y},\,{K}_{m}^{y}$), but scaled differently, using a separate parameter (${K}_{t}$). Furthermore, while this approach works for the study of one particular system, studying two or more systems together in an organism using this framework would also require understanding of the interactions and interdependences between the two systems in question, which are not always known.

Also, several other aspects of microbial evolution are beyond the scope of this framework. Through this framework, we cannot account for neutral mutations and their role in dictating the evolutionary dynamics of microbial populations^40^. This is due to the nature of framework, where every mutation alters the value of one of the chosen regulatory parameters, which implies, that every mutation will have some effect (large or small) on the steady state of the system variables and hence the fitness that is computed. The chance of a fully neutral mutation in the framework is generally low, except in some rare parameter regimes.

Additionally, our model works on the fundamental assumption that all mutations in the framework are single base substitutions such that the entire genome size and function remains constant^6,7^. Thus, our framework cannot capture the effects of insertions, deletions, and also horizontally acquired DNA and its role in dictating the evolutionary trajectory of the population, since it alters both the genome size and function.

Despite these limitations, however, our analysis does shed light on several interesting features associated with the DFE of beneficial and deleterious mutations in the lactose utilization system. Furthermore, the pre-existing thorough knowledge of the system physiology associated with the network allows us to capture the nature of epistatic interactions using the same mutational framework of altering model parameters. As newer models of gene regulation and metabolism at the genome scale become available^39,41–43^, it is conceivable that a framework such as the one presented in this study can be extended to the complete metabolic map of an organism. These features, we believe, will attract further theoretical and experimental work in this area.

## Supplementary information

Supplementary Data and Figures.
