# Supplementary material for: Distribution of fitness effects of mutations obtained from a simple genetic regulatory network model
Source: Sci Rep. 2019 Jul 8;9:9842. doi: 10.1038/s41598-019-46401-7 (PMC6614479; doi:10.1038/s41598-019-46401-7)
Supplement: Supplementary file 1 — Supplementary Data and Figures. [file 41598_2019_46401_MOESM1_ESM.docx]

**Supplement:**

**Distribution of fitness effects of mutations obtained from a simple genetic regulatory network model**

R. G. Brajesh^#^, Dibyendu Dutta^#^, and Supreet Saini*

Department of Chemical Engineering, Indian Institute of Technology Bombay

Powai, Mumbai – 400 076, India

* Corresponding Author. Email: [saini@che.iitb.ac.in](mailto:saini@che.iitb.ac.in); Phone Number: 91 22 2576 7216

^#^ Authors contributed equally to this work

**Table S1. List of parameters used in the model**

| Parameter | Parameter value | Unit | Reference |
| --- | --- | --- | --- |
| Rate of influx of lactose $\left( \boldsymbol{k}_{\boldsymbol{1}} \right)$ | 2148 | Min^-1^ | [^1^](#_ENREF_1) |
| Half saturation constant for lactose influx $\left( \boldsymbol{k}_{\boldsymbol{2}} \right)$ | 0.26 | mM | [^1^](#_ENREF_1) |
| Degradation rate of LacY $\left( \boldsymbol{k}_{\boldsymbol{d}\boldsymbol{1}} \right)$ | 0.65 | Min^-1^ | [^2^](#_ENREF_2) |
| Degradation rate of LacZ $\left( \boldsymbol{k}_{\boldsymbol{d}\boldsymbol{2}} \right)$ | 8.3e-2 | Min^-1^ | [^2^](#_ENREF_2) |
| Degradation rate of LacI $\left( \boldsymbol{k}_{\boldsymbol{d}\boldsymbol{3}} \right)$ | 0.023 | Min^-1^ | [^2^](#_ENREF_2) |
| Maximal activity of beta-galactosidase $\left( \boldsymbol{k}_{\boldsymbol{3}} \right)$ | 9540 | Min^-1^ | [^3^](#_ENREF_3)^,^[^4^](#_ENREF_4) |
| Half saturation constant for beta-galactosidase activity $\left( \boldsymbol{k}_{\boldsymbol{4}} \right)$ | 0.14 | mM | [^4^](#_ENREF_4) |
| Growth/Dilution rate of *E. coli* | 0.03 | Min^-1^ | [^1^](#_ENREF_1) |
| Basal expression level of LacZ and LacY $\left( \boldsymbol{Bas}_{\boldsymbol{1}} \right)$ (p1) | Between 0-1 | Min^-1^ |  |
| Basal expression level of LacI $\left( \boldsymbol{Bas}_{\boldsymbol{2}} \right)$ (p2) | Between 0-0.1 | Min^-1^ |  |
| Maximal promotor activity of lac operon $\left( \boldsymbol{k}^{\boldsymbol{y}} \right)$ (p3) | Between 0-200 | Min^-1^ |  |
| Half saturation constant for maximal promotor activity $\left( \boldsymbol{K}_{\boldsymbol{m}}^{\boldsymbol{y}} \right)$ (p4) | Between 0-0.1 | mM |  |
| Translation capacity of LacY over LacZ $\left( \boldsymbol{K}_{\boldsymbol{t}} \right)$ (p5) | 0-1000 |  |  |

**Table S2. List of 100 different parameter sets corresponding to same 0.001f*max* fitness.**

| **S.No** | $\boldsymbol{Bas}_{\boldsymbol{1}}$ | $\boldsymbol{Bas}_{\boldsymbol{2}}$ | $\boldsymbol{K}^{\boldsymbol{y}}$ | $\boldsymbol{K}_{\boldsymbol{m}}^{\boldsymbol{y}}$ | $\boldsymbol{K}_{\boldsymbol{t}}$ |
| --- | --- | --- | --- | --- | --- |
| 1 | 0.223541 | 0.093368 | 95.7761 | 0.053278 | 122.4404 |
| 2 | 0.674665 | 0.084297 | 127.1056 | 0.070087 | 366.1589 |
| 3 | 0.326483 | 0.041329 | 174.4581 | 0.097015 | 131.8305 |
| 4 | 0.119577 | 0.009737 | 153.6823 | 0.085688 | 58.32372 |
| 5 | 0.011801 | 0.062761 | 160.5109 | 0.08846 | 370.4239 |
| 6 | 0.868313 | 0.065948 | 124.0402 | 0.067633 | 669.5851 |
| 7 | 0.904999 | 0.04358 | 76.84871 | 0.042221 | 466.9224 |
| 8 | 0.779326 | 0.009424 | 162.24 | 0.089363 | 395.9491 |
| 9 | 0.536936 | 0.027722 | 172.7789 | 0.096509 | 13.1226 |
| 10 | 0.058753 | 0.071301 | 163.274 | 0.08829 | 877.2235 |
| 11 | 0.202082 | 0.086328 | 133.9146 | 0.073674 | 418.4712 |
| 12 | 0.924301 | 0.036174 | 150.8181 | 0.081292 | 973.6804 |
| 13 | 0.133393 | 0.07618 | 75.23207 | 0.04069 | 870.9965 |
| 14 | 0.799555 | 0.010882 | 152.1708 | 0.083227 | 582.8625 |
| 15 | 0.16029 | 0.02 | 43.60864 | 0.02415 | 236.2893 |
| 16 | 0.546291 | 0.067279 | 70.72223 | 0.038813 | 485.4908 |
| 17 | 0.805159 | 0.027725 | 176.4298 | 0.096472 | 587.3712 |
| 18 | 0.035118 | 0.068085 | 119.2371 | 0.065143 | 598.7672 |
| 19 | 0.270058 | 0.024783 | 95.52538 | 0.052032 | 681.5718 |
| 20 | 0.173783 | 0.047894 | 176.6548 | 0.098271 | 112.683 |
| 21 | 0.896239 | 0.095085 | 124.8044 | 0.069504 | 92.64079 |
| 22 | 0.463983 | 0.084754 | 172.4664 | 0.095635 | 202.3187 |
| 23 | 0.913496 | 0.002762 | 115.6387 | 0.063606 | 427.1195 |
| 24 | 0.0434 | 0.084531 | 95.64723 | 0.052882 | 274.1628 |
| 25 | 0.211554 | 0.044796 | 84.33765 | 0.046837 | 155.4234 |
| 26 | 0.77172 | 0.068442 | 177.9644 | 0.097367 | 565.7909 |
| 27 | 0.752148 | 0.091429 | 34.10109 | 0.018471 | 832.7745 |
| 28 | 0.820992 | 0.091242 | 77.86877 | 0.04305 | 283.1125 |
| 29 | 0.59566 | 0.094836 | 161.4013 | 0.087973 | 660.1548 |
| 30 | 0.686222 | 0.011926 | 159.1576 | 0.086624 | 699.2744 |
| 31 | 0.737361 | 0.019406 | 135.1313 | 0.073594 | 683.1227 |
| 32 | 0.431226 | 0.022954 | 8.439025 | 0.004576 | 791.1683 |
| 33 | 0.901729 | 0.072068 | 114.6421 | 0.062356 | 716.6707 |
| 34 | 0.084851 | 0.077848 | 64.79173 | 0.035999 | 130.5691 |
| 35 | 0.51358 | 0.088784 | 177.2835 | 0.095582 | 942.52 |
| 36 | 0.876164 | 0.083915 | 179.8059 | 0.098951 | 400.8314 |
| 37 | 0.832203 | 0.001669 | 180.4699 | 0.099475 | 356.5115 |
| 38 | 0.954845 | 0.062797 | 135.2991 | 0.073027 | 918.7913 |
| 39 | 0.858423 | 0.010636 | 131.8491 | 0.071876 | 653.5162 |
| 40 | 0.905138 | 0.047204 | 171.7494 | 0.094537 | 395.159 |
| 41 | 0.51484 | 0.007327 | 156.6759 | 0.085395 | 652.6609 |
| 42 | 0.585021 | 0.049615 | 85.37419 | 0.046931 | 424.9226 |
| 43 | 0.41582 | 0.055493 | 135.8164 | 0.07492 | 327.0474 |
| 44 | 0.855325 | 0.037555 | 169.3207 | 0.09176 | 808.0281 |
| 45 | 0.483836 | 0.020058 | 166.9988 | 0.091442 | 526.9752 |
| 46 | 0.420027 | 0.001858 | 145.3876 | 0.080253 | 308.5476 |
| 47 | 0.086327 | 0.07973 | 168.8092 | 0.092061 | 627.4794 |
| 48 | 0.314281 | 0.04391 | 43.72865 | 0.023808 | 675.027 |
| 49 | 0.940356 | 0.027633 | 138.7636 | 0.076862 | 219.2511 |
| 50 | 0.578431 | 0.084434 | 33.93326 | 0.018669 | 396.0416 |
| 51 | 0.449031 | 0.059444 | 161.0918 | 0.087582 | 711.097 |
| 52 | 0.274499 | 0.030882 | 149.4902 | 0.081015 | 793.0994 |
| 53 | 0.999004 | 0.084002 | 86.94608 | 0.047748 | 448.8134 |
| 54 | 0.649527 | 0.038724 | 160.2257 | 0.088602 | 257.2089 |
| 55 | 0.144433 | 0.044699 | 93.414 | 0.050239 | 990.7998 |
| 56 | 0.02892 | 0.045792 | 162.5205 | 0.088394 | 690.377 |
| 57 | 0.998263 | 0.032197 | 67.75741 | 0.036861 | 698.1059 |
| 58 | 0.102146 | 0.095007 | 148.6684 | 0.0818 | 380.18 |
| 59 | 0.2624 | 0.049555 | 79.17144 | 0.042628 | 959.9585 |
| 60 | 0.624063 | 0.029532 | 181.5028 | 0.098246 | 824.4738 |
| 61 | 0.493227 | 0.082742 | 148.9485 | 0.080508 | 860.9431 |
| 62 | 0.80666 | 0.070468 | 103.4643 | 0.055942 | 855.5152 |
| 63 | 0.152626 | 0.076963 | 173.3657 | 0.093422 | 934.7112 |
| 64 | 0.477314 | 0.05435 | 98.3809 | 0.054235 | 331.3143 |
| 65 | 0.389059 | 0.060305 | 96.64864 | 0.052857 | 544.0589 |
| 66 | 0.057021 | 0.008172 | 119.3966 | 0.066482 | 55.22035 |
| 67 | 0.226057 | 0.051972 | 160.6625 | 0.089473 | 51.97945 |
| 68 | 0.838139 | 0.044825 | 129.9427 | 0.071306 | 458.0492 |
| 69 | 0.098111 | 0.041075 | 178.5386 | 0.097404 | 603.1148 |
| 70 | 0.228364 | 0.004932 | 116.837 | 0.064556 | 261.9212 |
| 71 | 0.837392 | 0.022258 | 104.4691 | 0.058171 | 62.22654 |
| 72 | 0.559293 | 0.085223 | 86.72452 | 0.04722 | 660.2148 |
| 73 | 0.741495 | 0.022429 | 160.5426 | 0.088249 | 407.2632 |
| 74 | 0.590018 | 0.015828 | 141.4424 | 0.07647 | 847.0611 |
| 75 | 0.130951 | 0.005582 | 154.313 | 0.084443 | 518.6324 |
| 76 | 0.517125 | 0.074212 | 41.58607 | 0.023043 | 186.712 |
| 77 | 0.414358 | 0.072576 | 96.70946 | 0.05291 | 527.0992 |
| 78 | 0.705437 | 0.075771 | 112.0676 | 0.06025 | 995.3368 |
| 79 | 0.082476 | 0.097669 | 161.1189 | 0.089522 | 104.2485 |
| 80 | 0.254397 | 0.071678 | 47.86276 | 0.026198 | 512.3638 |
| 81 | 0.016557 | 0.084082 | 145.6029 | 0.080134 | 360.4389 |
| 82 | 0.150642 | 0.067856 | 133.5225 | 0.073848 | 228.0817 |
| 83 | 0.5632 | 0.0138 | 181.7751 | 0.099762 | 442.604 |
| 84 | 0.118963 | 0.065076 | 131.2173 | 0.071569 | 601.9884 |
| 85 | 0.720722 | 0.074114 | 139.59 | 0.07687 | 352.3955 |
| 86 | 0.180929 | 0.027404 | 104.3196 | 0.057576 | 283.2366 |
| 87 | 0.535517 | 0.029894 | 112.2319 | 0.061755 | 369.7843 |
| 88 | 0.766667 | 0.029643 | 183.8534 | 0.098932 | 967.4879 |
| 89 | 0.556338 | 0.099502 | 182.2707 | 0.098634 | 815.6573 |
| 90 | 0.178158 | 0.0602 | 166.5394 | 0.089973 | 852.0192 |
| 91 | 0.775773 | 0.050442 | 106.1058 | 0.057254 | 891.6156 |
| 92 | 0.91334 | 0.053624 | 147.968 | 0.082198 | 113.5885 |
| 93 | 0.962936 | 0.073265 | 157.4469 | 0.087349 | 149.614 |
| 94 | 0.85225 | 0.076847 | 162.604 | 0.090283 | 125.099 |
| 95 | 0.435123 | 0.052884 | 67.08712 | 0.036352 | 772.6484 |
| 96 | 0.479683 | 0.030091 | 89.72974 | 0.049743 | 160.8913 |
| 97 | 0.356045 | 0.011803 | 181.6884 | 0.097617 | 996.2791 |
| 98 | 0.679347 | 0.01152 | 94.69353 | 0.052638 | 87.11677 |
| 99 | 0.936174 | 0.020159 | 137.5248 | 0.07607 | 224.9572 |
| 100 | 0.540881 | 0.053415 | 33.40112 | 0.018191 | 635.919 |

**P0** {p1, p2, p3, p4, p5} 🡪 *f0*

**P0*** {p1, p2, p3*, p4, p5} 🡪 *f0**

**Introduce beneficial mutation in p3**

**P_M_** {p1_M_, p2, p3, p4, p5} 🡪 *f_M_*

**P_M_*** {p1_M_, p2, p3*, p4, p5} 🡪 *f_M_**

**Introduce beneficial mutation in p3**

Δ*f* = *f0* – f0*

Δ*f** = *f_M_* - f_M_*

**Supplementary Figure S1.** Study of epistasis between beneficial mutations. A beneficial mutation pi (shown in parameter 3) is introduced in a parameter set P0 to obtain parameter set P0*. The set P0 corresponds to fitness *f0*, and P0* corresponds to *f0**. The benefit conferred by the mutation is represented by *Δf.* The same mutation pi (in parameter 3) is introduced in set P_M_, which is carrying a beneficial mutation in one of the other parameters (in parameter p1, as shown in the Figure above). The benefit conferred by the mutation pi in this set is represented by Δ*f*.* For each beneficial mutation pi, the ratio Δ*f/*Δ*f** is calculated for about 4000 sets (each carrying a distinct beneficial mutation – randomly spread over the remaining four parameters).












**Supplementary Figure S2. Epistatic interactions between beneficial mutations.** The analysis in Figure 6 is repeated for four distinct mutations in each of the five parameters. We note that the spread of the ratio *Δf/Δf** is qualitatively identical in each of the five cases, and therefore conclude that the spread is independent of the precise mutation introduced in the parameter.





**Supplementary Figure S3.** The clusters observed in Figures 6 and S2 are because of the background in which a beneficial mutation pi is introduced. For instance, as shown in Figure above, when the background beneficial mutation is introduced in parameter p3 the spread of ratio Δ*f/*Δf* looks like the leftmost data (black). When the ratio is analyzed for sets which carry an additional beneficial mutation in p1, the spread is as shown in the second data (red). Similarly, the ratio of beneficial effects because of a mutation in p3, when introduced in a set carrying a beneficial mutation in p2, p4, or p5 is as represented in blue, pink, and green respectively. Therefore, the clusters seen in Figure 6 and S2 are due to the parameter in which the parameter set is carrying an additional benefit in.


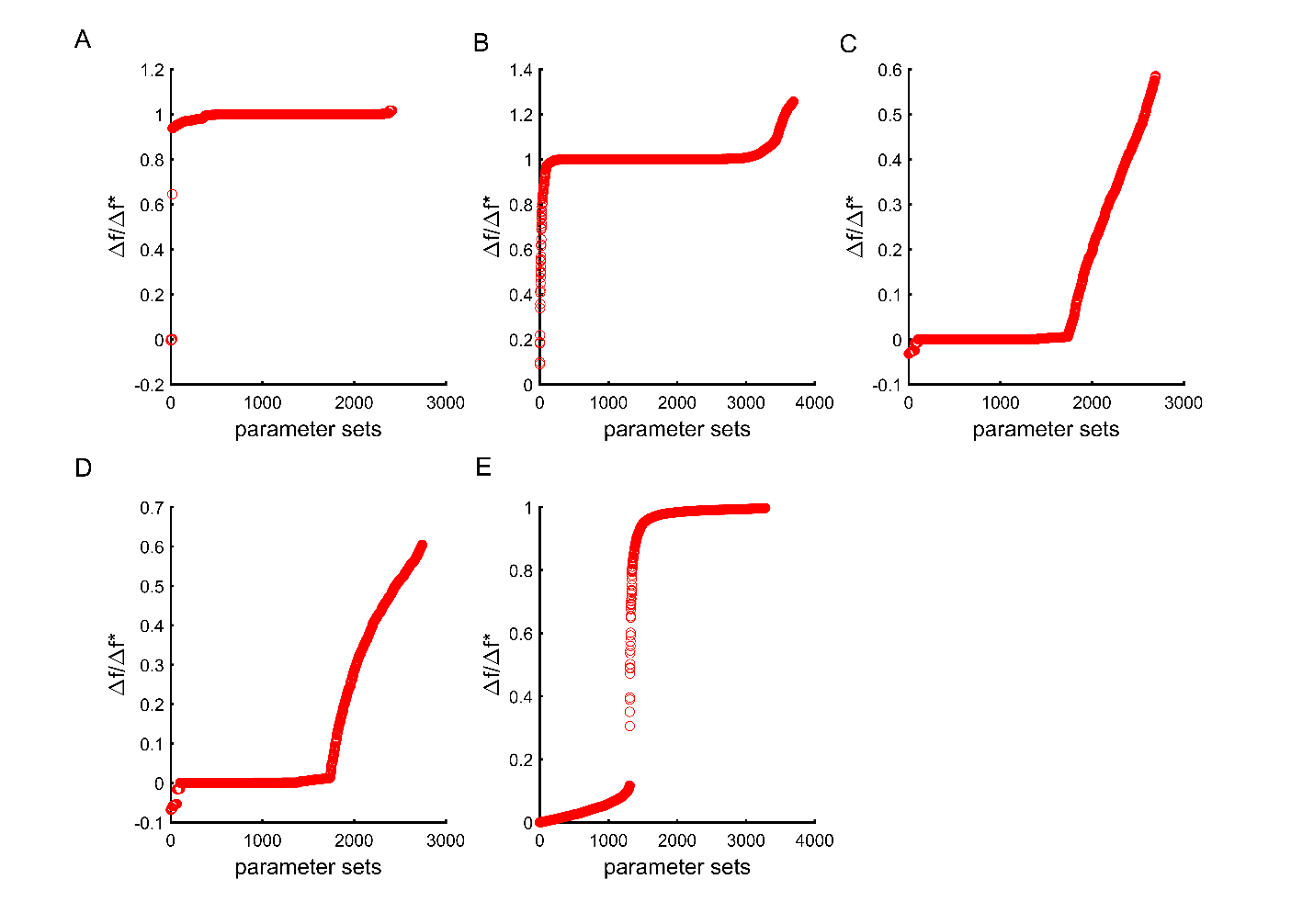


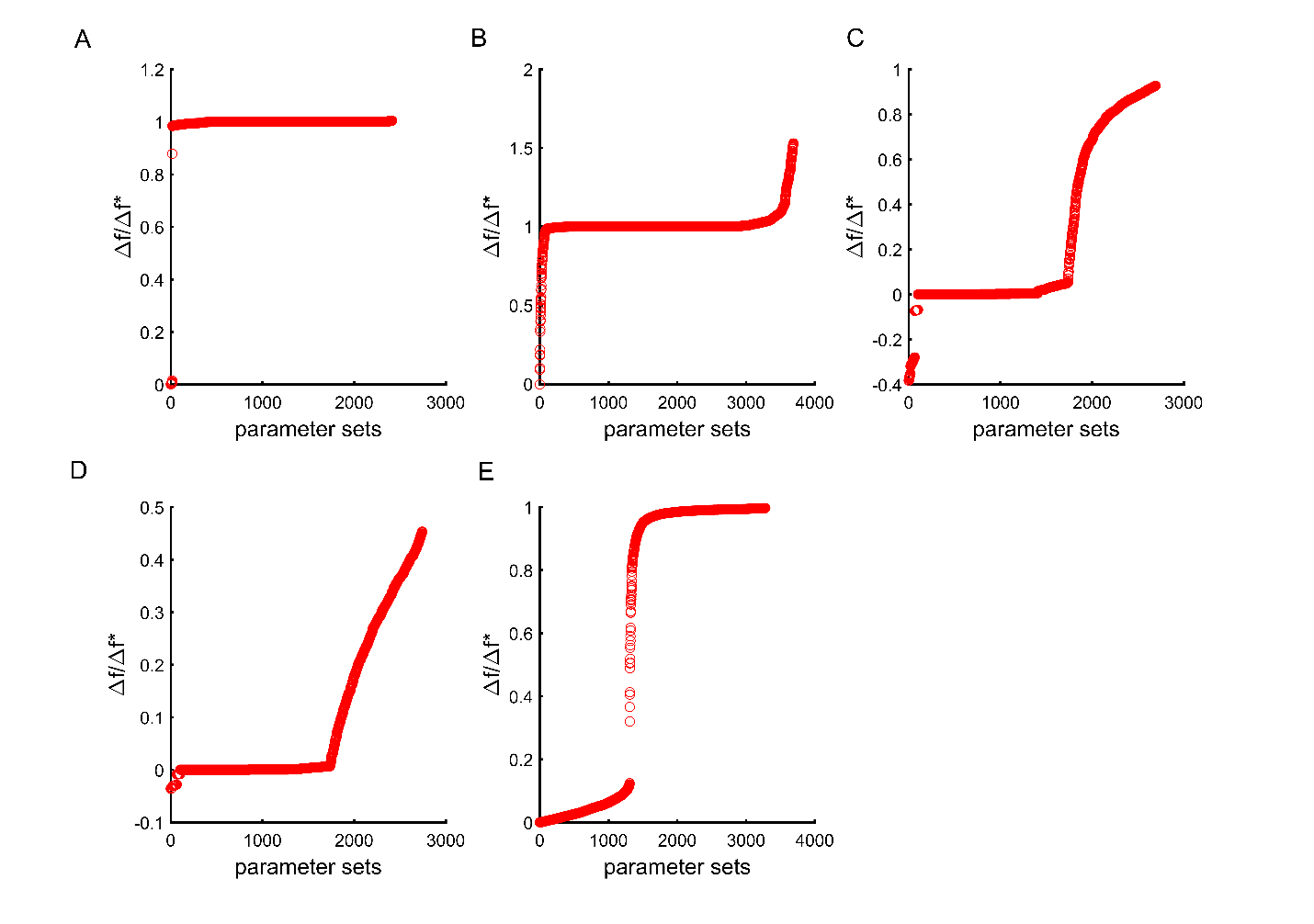


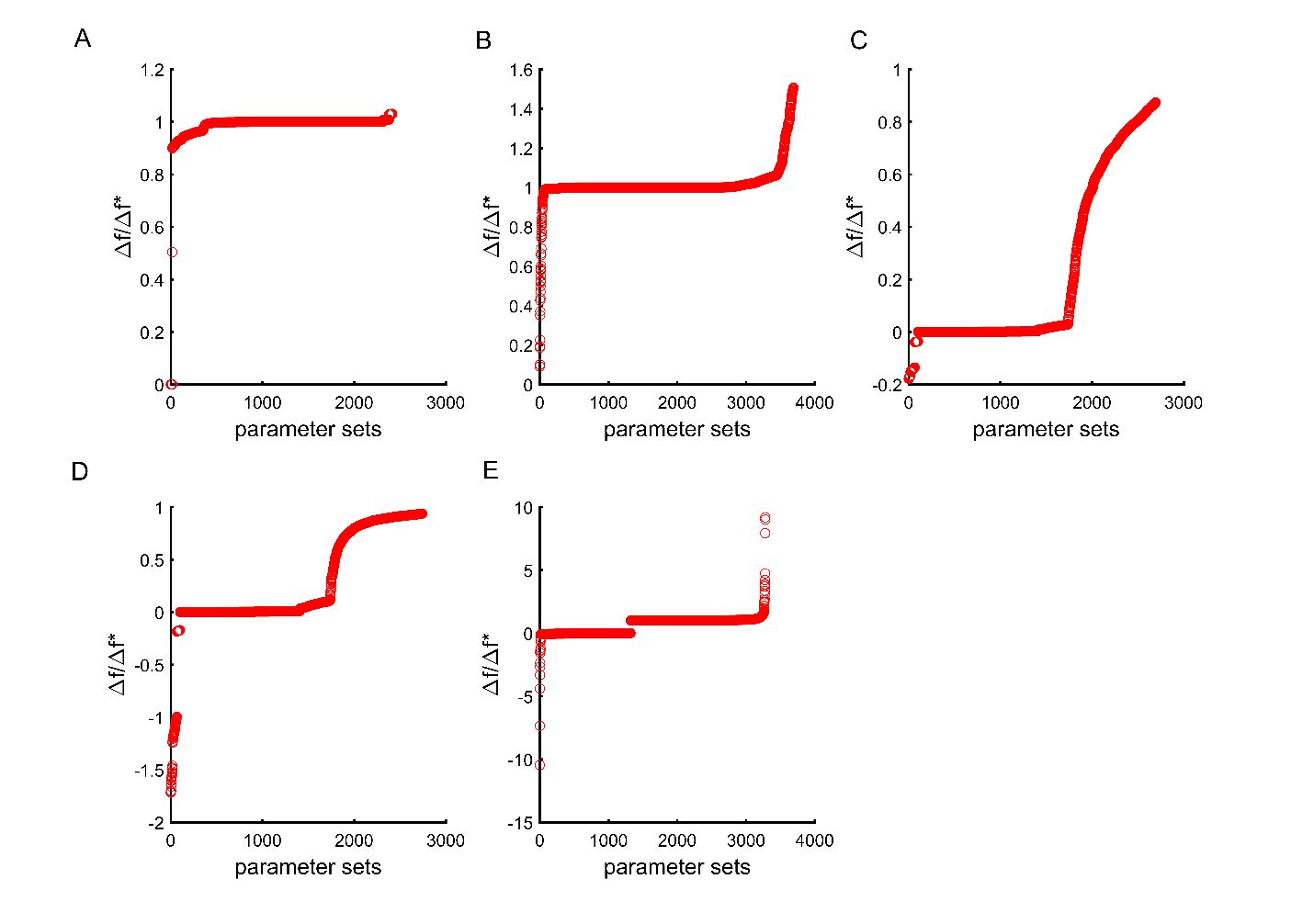


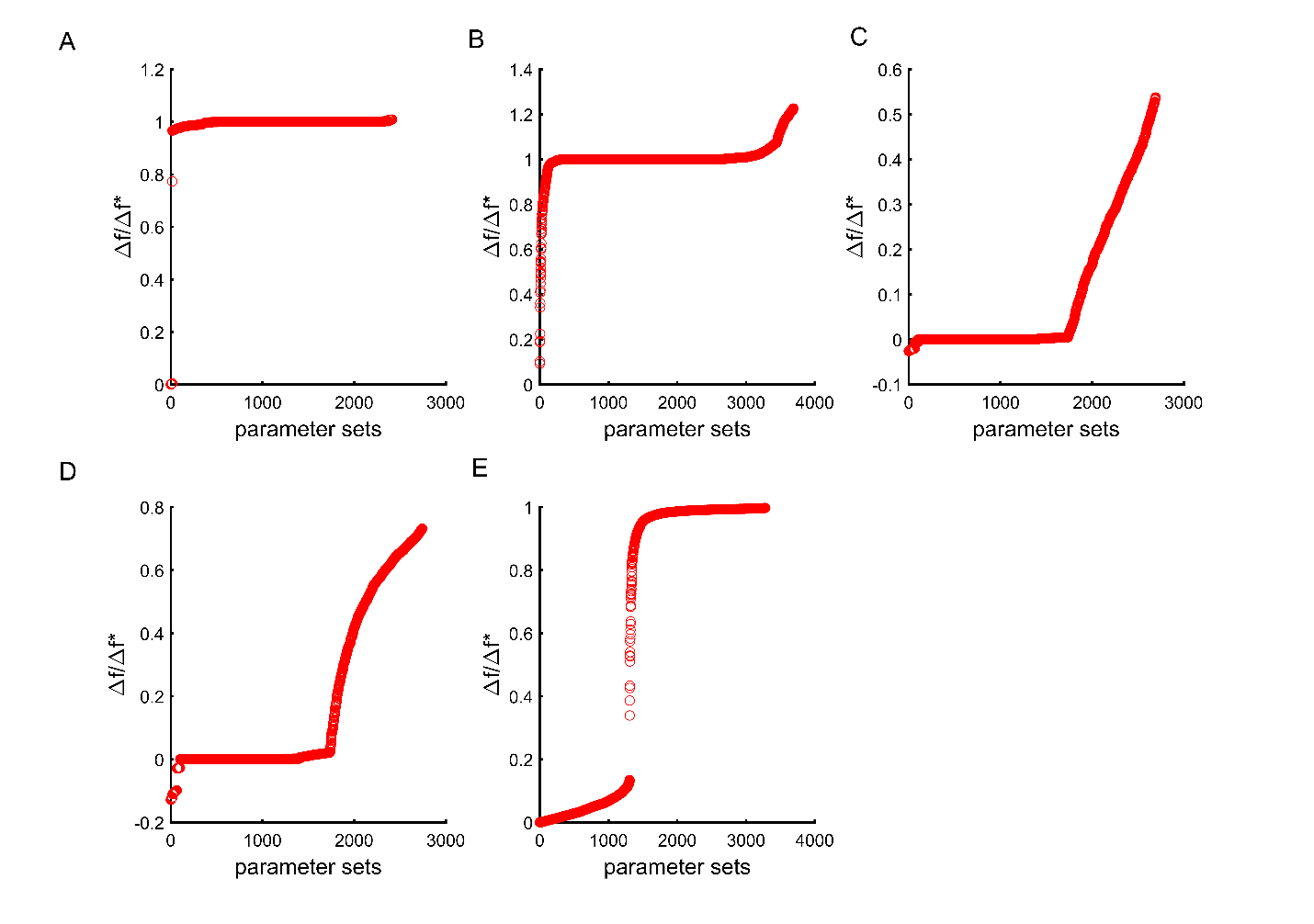


**Supplementary Figure S4. The benefit conferred by a mutation decreases as the fitness of the parameter set in which it is introduced increases.** The above four charts represents the ratio of fitness effects due to four distinct mutations (in addition to the mutations portrayed in Figure 7) in each of the five parameters involved. The X-axis represents the parameter sets arranged in the increasing order of their initial fitness. The data exhibits the qualitative agreement between the trends observed for different mutations for each of the five parameters.

**Sensitivity Analysis**

**Methodology**

Sensitivity Analysis is performed on any model to test the variability in the outcome based on variation in the input parameters. In our work here, many of the parameters used in the making of our lactose utilization system are molecular chemical constants which are accurately determined and hence we can be relatively unconcerned about variability in these parameters causing variation in the model outcomes.

In our case, it may only be necessary to test the sensitivity of the parameters which we consider to be genetically tunable, since they account for the fitness differences in the model outcome.

The parameters are - $Bas_{1}, Bas_{2}, k^{y}, K_{m}^{y},$ and $K_{t}$, which are referred to as p1, p2, p3, p4, p5 respectively.

Our model is built using a “wild type” parameter set, and then we vary the chosen parameters within an identified range, and then we obtained different parameter sets based on the final fitness values. We used 100 parameter sets for 0.001fmax, and 80 parameter sets for 0.5fmax, and used these for estimation of Distribution of Fitness Effects (DFE).

In our sensitivity analysis we chose each of the parameter sets, and vary each of the parameters across their entire range and plot (1) the resulting fitness and (2) the percentage change in fitness starting from (i) 0.001fmax and (ii) 0.5fmax.

In each plot we report the fitness and the percentage change in fitness for each of the parameter sets at the same fitness, when we vary a single parameter at 10% intervals. We represent the data points in the form of violin plots at each of the specific parameter values.


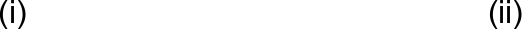

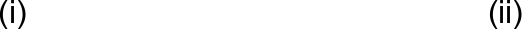


A

B

C


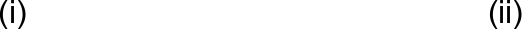

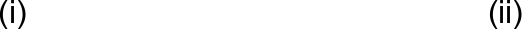

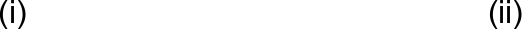


DC

E

**Supplementary Figure S5. Sensitivity Analysis for parameter sets with fitness 0.001fmax**

Panels (A)-(E) represent the sensitivity analysis done for each of the 5 parameters - $Bas_{1},$ $Bas_{2}, k^{y}, K_{m}^{y},$ and $K_{t}$ respectively. For each panel (i) represents the actual fitness values as computed using our cost-benefit framework, and (ii) represents the percentage change in fitness due to the change in the parameter value.


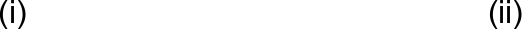

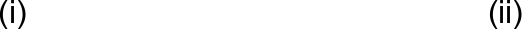


A

B

C


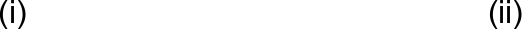

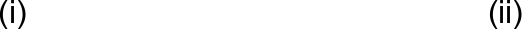

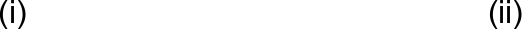


DC

E

**Supplementary Figure S6. Sensitivity Analysis for parameter sets with fitness 0.5fmax**

Panels (A)-(E) represent the sensitivity analysis done for each of the 5 parameters - $Bas_{1},$ $Bas_{2}, k^{y}, K_{m}^{y},$ and $K_{t}$ respectively. For each panel (i) represents the actual fitness values as computed using our cost-benefit framework, and (ii) represents the percentage change in fitness due to the change in the parameter value.

**Supplement References.**

1 Wong, P., Gladney, S. & Keasling, J. D. Mathematical model of the lac operon: inducer exclusion, catabolite repression, and diauxic growth on glucose and lactose. *Biotechnology progress* **13**, 132-143, doi:10.1021/bp970003o (1997).

2 Yildirim, N. & Mackey, M. C. Feedback regulation in the lactose operon: a mathematical modeling study and comparison with experimental data. *Biophysical journal* **84**, 2841-2851, doi:10.1016/S0006-3495(03)70013-7 (2003).

3 Huber, R. E., Kurz, G. & Wallenfels, K. A quantitation of the factors which affect the hydrolase and transgalactosylase activities of beta-galactosidase (E. coli) on lactose. *Biochemistry* **15**, 1994-2001 (1976).

4 Huber, R. E., Pisko-Dubienski, R. & Hurlburt, K. L. Immediate stoichiometric appearance of beta-galactosidase products in the medium of Escherichia coli cells incubated with lactose. *Biochemical and biophysical research communications* **96**, 656-661 (1980).
